# Prediction of biomarkers of oral squamous cell carcinoma using microarray technology

**DOI:** 10.1038/srep42105

**Published:** 2017-02-08

**Authors:** Guang Li, Xian Li, Meng Yang, Lvzi Xu, Shixiong Deng, Longke Ran

**Affiliations:** 1 Department of Bioinformatics, Chongqing Medical University, Chongqing, China; 2 College of Medical Informatics, Chongqing Medical University, Chongqing, China; 3 Research Department, Children Hospital of Chongqing Medical University, Chongqing, China

## Abstract

Microarray data is used to screen the genes of oral squamous cell carcinoma (OSCC). Microarray data of OSCC and normal tissues were downloaded from GEO database and analyzed with Benjamini-Hochberg (BH) method. Differentially expressed genes (DEGs) were then uploaded on DAVID database to process enrichment analysis. Target genes were finally chosen for verification experiment *in vitro* and *in vivo*. 78 DEGs were selected from 54676 genes, including 46 up- and 32 down- regulation. GO term showed that these genes were related to epidermal growth (biological processes), extracellular region (cellular components) and cytokines activity (molecular function). Protein network interaction demonstrated that OSCC was closely allied to the five key genes including CXCL10, IFI6, IFI27, ADAMTS2 and COL5A1, which was consistent with the RT-PCR data. High-expressed gene CXCL10 was chosen for further cell experiment, and the results indicated that CXCL10 can promote the proliferation, migration and invasion of normal cells and inhibited the cancer cells after si-RNA transfection. Moreover, it has been proven that CXCL10 was possibly related to the occurrence and development of OSCC. Understanding the regulation of OSCC expression will shed light on the screening of cancer biomarker.

1.6 million people in the world suffered from head and neck squamous cell carcinoma (HNSCC)^1^, and 330 thousand people died every year. About half were oral squamous cell carcinoma (OSCC)^2^, which was a highly aggressive head and neck tumor and prone to local recurrence and metastasis^3^. The development of OSCC was a long-time, multi-stage and multi-factor process, and many regulatory factors were involved in cell carcinogenesis^4^. However, the detailed molecular mechanism of this cancer was still unclear. Previous studies^5^ have reported that consecutive reactions were formed through various abnormal expressed genes, and the gene expression profile was the key to find pathological mechanism of OSCC^6^. Traditional methods of gene expression analysis, such as Northern-Blotting technology^7^, were mainly concerned with single or several genes. However, the interaction effects can not be found from multiple genes.

Microarray, as a novel technology, represented that thousands of DNA probes corresponding to target genes were placed on a tiny chip, which can determine the gene expression from the samples^8^,^9^. The method was mainly applied to comparison difference between cancer and normal tissues, different subtypes of cancer, or patients with different prognosis, and so on^10^. From these studies, people could gradually understand the mechanism of diseases, master effective methods for the diagnosis and detection of disease, and predict patients’ prognosis, which showed a great significance for the diagnosis and treatment of the disease in the future.

In 1999, T.Golub^11^ firstly focused on cancer classification using microarray, and the results represented that gene expression data obtained from oligonucleotide microarray, including acute myeloid leukemia (AML) and acute lymphoblastic leukemia (ALL) were successfully separated, and DEGs were listed in the two diseases, which revealed feasibility and potential of microarray technology in cancer classification. A new leukemia subtype was followed and divided through clustering analysis with these gene expression data. Recently, microarray technology has been widely applied in cancer research, including various types of leukemia^12^, lung cancer^13^, and prostate cancer^14^, which provides a new strategy of pathogenesis of cancer on the molecular level.

In this study, we aimed to analyze the differentially expressed genes (DEGs) using the gene expression profile analysis between OSCC and normal tissues by microarray technology. DEGs were selected and regarded as the genes related to OSCC development, and verification experiments were used to understand the pathogenesis of oral cancer. In addition, further molecular mechanism and genetic characteristics of OSCC were discussed to explore a potential gene therapy of OSCC.

## Materials and Methods

### Gene screening

#### Data preparation

From GEO data searching, a total of 141 OSCC affimetrix literature were documented to identifiy, 23 studies were retained after removing duplicated records. 8 articles were excluded according to the title and sample names, and the remained 15 full-text articles were assessed for eligibility. Over half of these data were not passed for containing null values or the median-centered across samples was non-zero, which was not suitable for comparison. After that, 7 microarry datasets were finally recuited in analysis. These GSE datasets were downloaded from NCBI database (GEO http://www.ncbi.nlm.nih.gov/geo/), which can be detected by Affymetrix Human Genome Array Platform. All the GSE samples including OSCC and normal tissue two groups, we converted the data to (log)_2_(ratio) format and RMA expression software was applied to carry on data normalization, data transformation and quality control to guarantee reliable data in subsequent analysis^15^.

#### Differential expression analysis

R (3.1.1) limma package and Benjamini-Hochberg (BH) method^16^ was introduced for gene screening (P < 0.01), and differences between two groups were examined for more than 3 times. DEGs were then clustered by Cluster 3.0 and visualized with Java TreeView. These differential genes were uploaded to DAVID^17^ database to process function enrichment analysis and pathway analysis. Finally, with the help of STRING^18^, the probe number of DEGs was converted into protein, and the co-expression graph of protein network was obtained.

### Validation experiment

#### Sample preparation

12 cases of OSCC were collected from Oral Hospital of Chongqing Medical University, and 6 normal oral tissues were taken as controls. OSCC and normal cell lines were purchased from Chongqing Manuik Company, and cultured in MEM/EBSS medium containing 10% fetal bovine serum (FBS).

#### RT-PCR experiment

RNA samples were firstly extracted from OSCC and normal tissues, then superscript III kit (Invitrogen) were used to reverse transcription. Each sample was amplified with differentially expressed gene and relarant gene, and primers were designed according to the target genes. The scramble siRNA sequence was employed as the control transfection for both normal cell and OSCC cells.

#### CXCL10 siRNA transfection

OSCC cells were cultured in cell plate (6 holes) after 12 to 24 h, washed twice with serum-free MEM and stored in incubator. CXCL10 siRNA solution was added into the plate with 6 holes, blended and cultured after 4 to 6 h, then replaced with complete medium containing 10% FBS.

#### MTT method

Cells suspension (1 × 10^6^/ml) were collected after 48-hour transfection. Each hole was added with 20 μl 0.5% MTT solution, cultured for 4 h. The supernatant was centrifuged and drained, and 150 μl dimethyl sulfoxide was added in each hole, shaked on bed with low oscillation speed for 10 min to dissolve the crystals. Absorbance value of each hole was determined with enzyme-linked immune reader (OD 570 nm). At the same time, zero adjusting holes were contained with medium, MTT, dimethyl sulfoxide, control hole including cells, same concentration of drug dissolution medium, culture solution, MTT, and dimethyl sulfoxide, each group setting with 4 holes. Cell survival rate = (processed cells absorbance/ control group absorbance) × 100%, and the growth curve was drawing.

#### Wound healing experiment

After CXCL10 siRNA transfection, a linear wound was made by scraping a nonopening Pasteur pipette across the confluent cell layer. Cells were washed with PBS to remove detached cells and debris and incubated in serum-free medium. Images were observed with inverted microscope camera and analyzed with MATLAB software to measure the size of wounds at the indicated times. Gap fusion rate = (0 h gap width ~ 12 h gap width)/0 h gap width.

#### Transwell experiment

The upper room of Transwell was filled with 20 μl Matrigel and MEM (1:2), reacted in 37 °C for 30 min to form gel. The other room was added 500 μl medium containing serum. 200 μl of cell suspension were prepared, and 3 × 10^4^ cells were added in the upper room for 48 h, then fixed with paraformaldehyde for 3 min, followed by crystal violet staining for 5 min. Cells were observed under inverted microscope. 3 visions were selected randomly and the number of transmembrane cells was counted in each field.

#### Statement

The use of human oral tissue samples were approved with Chongqing Medical University (CQMU), all experiments were performed in accordance with relevant guidelines and regulations, all methods were carried out in accordance with relevant guidelines and regulations, and all experimental protocols were approved by CQMU. Informed consent was obtained from all subjects.

### Statistical methods

Mean and standard deviation (SD) values were calculated for each group of data. Analysis of variance (ANOVA) was performed to detect whether a significant difference (p < 0.05, n ≥ 3) existed between groups. The Holm t-test was used to identify any differences.

## Results

### Data profile

In this study, we collected a total of 7 gene expression profile datasets from previously published articles about OSCC. We transformed the GSE Series Matrix files(cel), platform sets, and annotation files (cdf) from the gene Expression Omnibus(GEO). We chose 8 normal data and 8 cancer data in each GSE dataset, including 112 array samples in total. According to RMA, normalization data were confirmed with high quality and can be used for further analysis. Details of all data sets were summarized in Table 1. The oral cancer tissues were performed as experiment group (C), while normal tissues were regarded as control (N).

### Differential expression analysis

**Figure 1:**
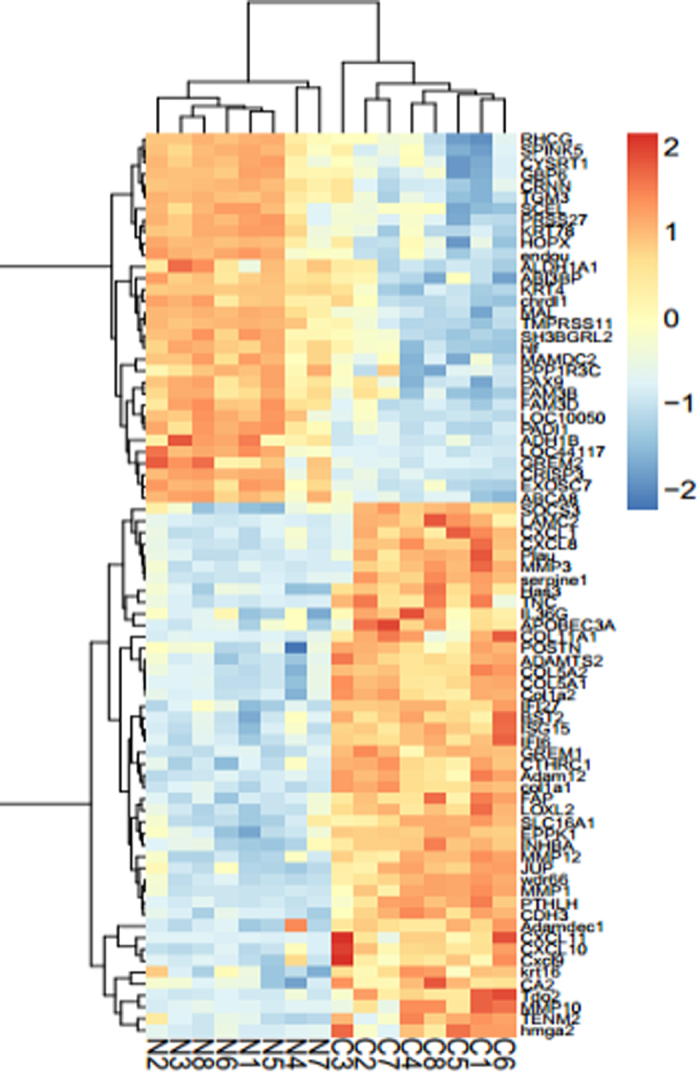
Hierarchical clustering heat map of DEGs by normal tissues (N) and cancer tissues (C) in OSCC.

For data precision and consistency, we use the BH method to dispose the data and adjust p-value to correct the data, FC was declared to be at least 3 fold and p < 0.01 was regarded as significant. Compared to normal tissues, 78 DEGs were selected from all the 7 datasets, including 46 up-regulated genes and 32 down-regulated genes. Figure 1 showed the heatmap of different expression of these genes.

### Enrichment analysis and pathway analysis

**Figure 2:**
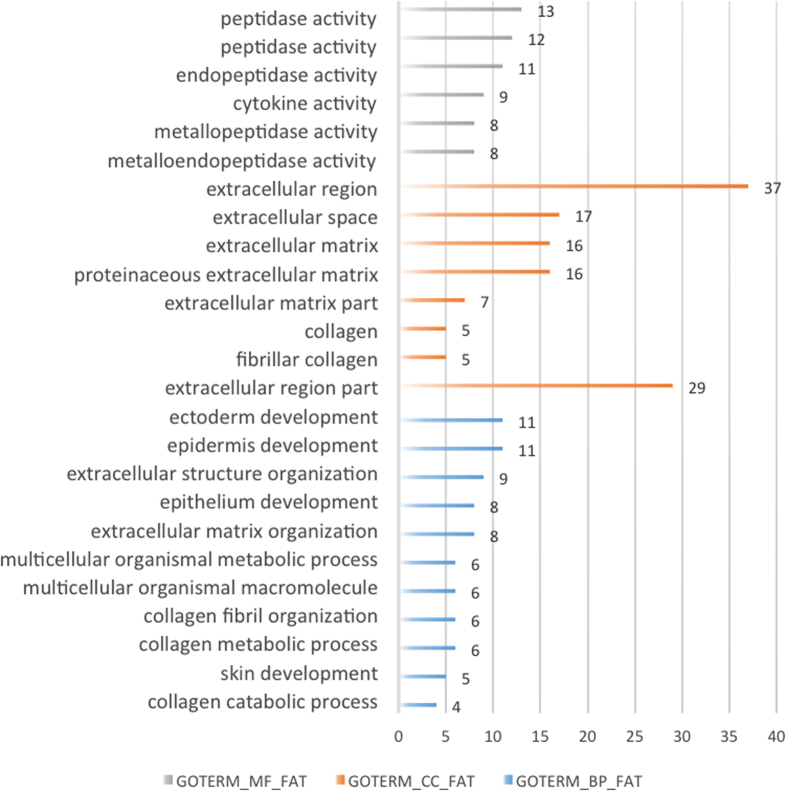
Biological process (BP), molecular function (MF) and cellular component (CC) of DEGs by function enrichment in PPI network.

Enrichment analysis were performed with DAVID to further explore the function of DEGs. GO showed that the DEGs were related to epidermal development, biofouling and other biological processes (BP), molecular structure activities and actin binding molecular function (MF), cell components (CC) with extracellular region (Fig. 2). KEGG pathway revealed that pathways including extracellular matrix interactions, small cell lung cancer and focal adhesion were mainly affected.

### Protein-protein interaction analysis

**Figure 3:**
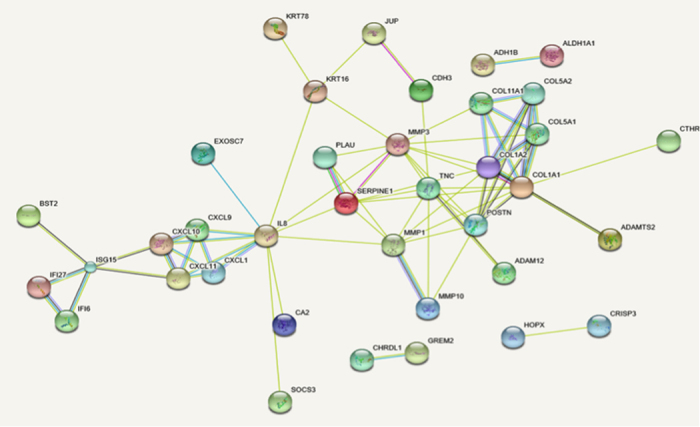
Protein-protein interaction (PPI) network of DEGs by STRING. The interaction score was set to high confidence (0.720).

All the gene probes were successfully translated to correspond expressed protein. Through uploading the multiple proteins on STRING, we obtained total 74 proteins identified as human, half of which were associated with each other, while the rest proteins were isolated. Then, a further analysis of protein co-expression network image (Fig. 3) were carried out and 5 key genes from the most network binding nodes were found including CXCL10, IFI6, IFI27, ADAMTS2 and COL5A1, which were all up-regulated in the mentioned expression results.

### RT-PCR

**Figure 4:**
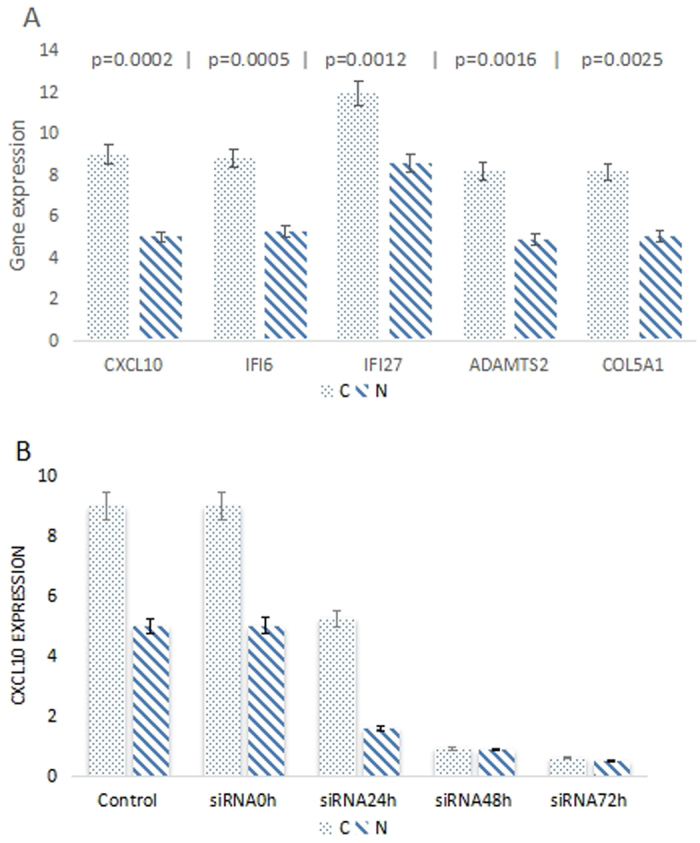
(**A**) Five target genes expression in normal and cancer tissues, the fold change of CXCL10 was higher among these genes; (**B**) CXCL10 expression at different transfection time (0–72 h), the scramble siRNA sequence was employed as control, at 48 h both cells showed lower expression.

The expression of five genes were verified in tissues using RT-PCR. As shown in Fig. 4, the expression of these genes in OSCC tissues were all significantly higher than normal tissues. The log_2_ (fold change) between normal and cancer was calculated. Among CXCL10 (3.99), IFI6 (3.57), IFI27 (3.35), ADAMTS2 (3.30) and COL5A1 (3.10), CXCL10 showed the highest fold and was chosen as the target gene in our further validation experiment. Then, OSCC and normal cell lines were transfected with CXCL10 siRNA and continuous observations were obtained. Figure 4 presented that the expression of CXCL10 significantly inhibited in two cell lines and reached the maximum in 48 h.

### Cell proliferation assay by MTT

**Figure 5:**
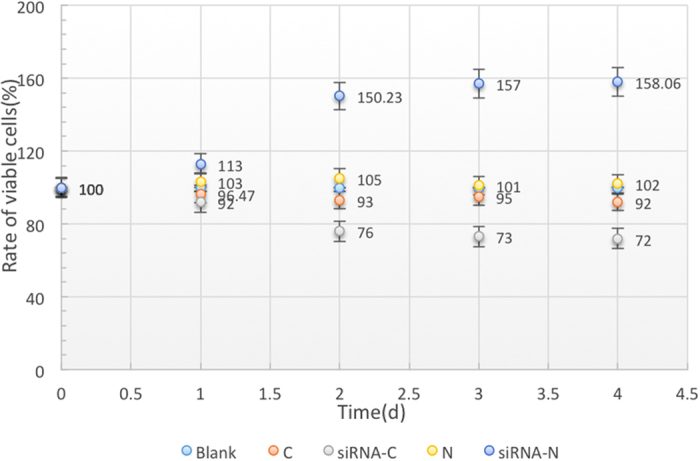
Rate of viable cells after different days (0–5d) siRNA transfection in both normal and cancer cells. siRNA-mediated CXCL10 promoted normal cells and inhibited cancer cells proliferation.

According to Fig. 5, cell proliferation was decreased significantly in cancer cells with low-expressed CXCL10. In contrast, after CXCL10 siRNA transfection for 48 h, the normal cells showed a growth of blowout, and the relative survival rate gradually increased, suggesting that differences were significant before and after transfection experiment (P < 0.05).

### Cell migration ability by wound healing test

**Figure 6:**
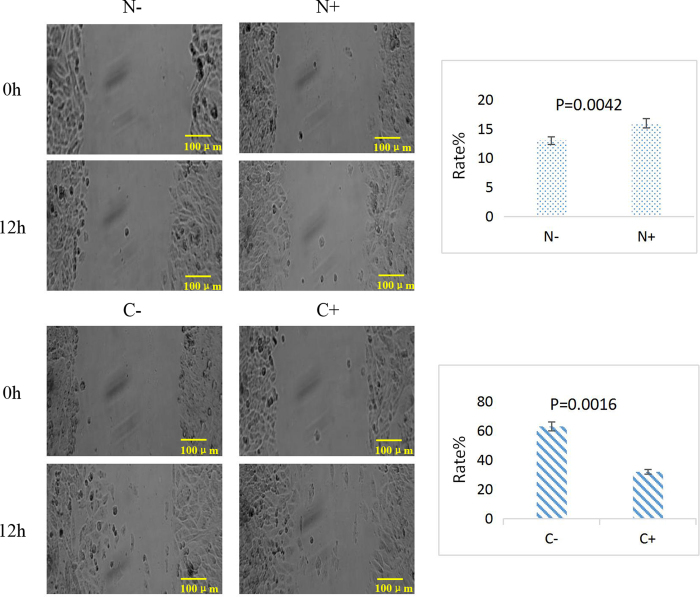
Wound healing experiment (100 μm) with CXCL10 siRNA transfection (0–12 h). Data and representative images were shown for normal and cancer cells. Bar charts showed the migration rate of each treatment.

The wound healing rate of cell lines in 0 h and 12 h were calculated (Fig. 6). Results showed that the rate of cancer cells was slower after transfection, while the rate of siRNA CXCL10 in normal cells was significantly increased (P < 0.05).

### Invasion ability by Transwell assay

**Figure 7:**
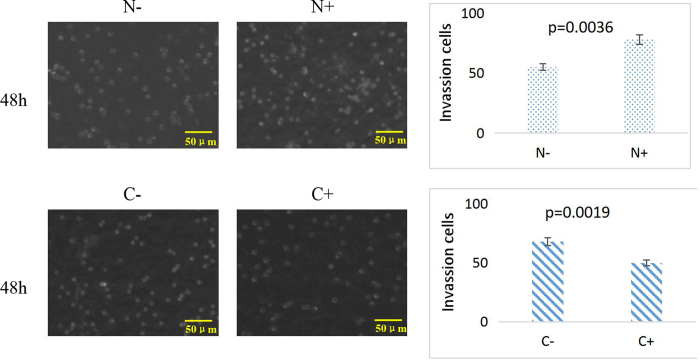
Transwell assays (50 μm) of normal and cancer cells with CXCL10 siRNA (0–48 h). The numbers of invading cells were counted, and a significant difference (P < 0.05) was observed.

The image was treated with MATLAB software and accessed by grey value. According to the random counting views of Transwell experiment showed that after transfected with siRNA CXCL10 for 48 h, normal cells appeared weaker invasion ability with non-transfected normal cells. On the contrary, the number of cancer cells was significantly increased in 48 h transfection (Fig. 7). A significant difference was observed in both cell lines and the numbers of migrating cells were counted (P < 0.05).

## Discussion

In this study, we downloaded microarray data from GEO, and finally found 78 DEGs through bioinformatical methods, including 46 up-regulated genes and 32 down-regulated genes, which were involved in transcription, apoptosis, anti-apoptosis, stress, RNA metabolism and so on. The diverse function made it possible that we could find the target genes of cancer and reveal molecular mechanism. The maximum expression genes reached 7.9(MMP1), 5.7(PTHLH) and 5.3(MMP3), and these genes were also closely connected each other in enrichment, clustering, protein co-expression network and KEGG pathway. Moreover, these gene links could be found in other cancers^19^, which were mainly related to gene transcription process. Recognition of the data enabled us to consider the further expansion of signal transduction pathway.

From all the genes, CXCL10, IFI6, IFI27, ADAMTS2 and COL5A1 were involved in the co-expression network, especially, CXCL10 was a up-regulated gene which linked the most number of nodes. However, the molecular mechanisms by which CXCL10 regulated cancer cell proliferation and invasion remained unclear. CXC chemokine ligand, also known as interferon inducible protein, can regulate immune response, angiogenesis, cell apoptosis, cell cycle and cell proliferation. CXCL10 20 was usually associated with the process from tumor occurrence, development and therapy to prognosis of cancer. Previous studies found that CXCL10 was a novel candidate oncogene which may be a therapeutic target for several types of human cancer. It had a dual effect on tumor, inhibition^21^ and promotion^22^.

It has been known that CXCL10 could directly combine with chemokine receptor3 (CXCR3) and have biological effect in cancer behaviors such as anti-proliferation. Giuliani^23^ showed that CXCL10 can affect the biological characters of myeloma through autocrine modes of action. It combined with CXCR3B receptor and significantly inhibited cell proliferation. CXCL10 activates CXCR3A receptors on myeloma cells, which attenuated apoptosis mediated by FAS (CD95). Besides, CXCL10 can promote cancer cell apoptosis by interfering oncogene expression. Zhang’s^24^ research presented that CXCL10 can inhibit the expression of HPV oncogene E6 and E7 and promoted the p53 expression, which finally caused Hela cell apoptosis. These findings provide evidence that the regulation of CXCL10 may play an important role in tumorigenesis. However, the abnormal expression of CXCL10 and its possible carcinogenesis in OSCC have not been addressed previously.

In this study, we detected changes in the expression of CXCL10 in cancer which was significantly higher than that of normal tissues. Further cell experiment indicated that CXCL10 expression obviously declined in cancer cells after its siRNA transfection, which inhibited the cancer cell proliferation and in turn promoted normal cell growth. Moreover, wound healing experiment showed normal cells speed up with low expressed CXCL10, while cancer cells demonstrated an obvious decline. In Transwell invasion experiment, the number of cancer cells through Transwell room decreased sharply while normal cells increased. These results indicated that the inhibition of CXCL10 can weaken the proliferation, migration and invasion ability of tumor, which suggested that CXCL10 abnormal expression may relate to the occurrence and development of oral cancer.

## Conclusion

In summary, bioinformatic methods were used to analyze the microarray data of OSCC, and 72 genes were finally screened out including 46 up-regulated genes, and 32 down-regulated genes. The results of RT-PCR on five significantly DEGs were consistent with theoretical analysis in 12 cases of OSCC and normal tissues, which expression changed by at least 3-fold. We performed cell experiment on CXCL10, whose gene expression reached the lowest after 48 hours transfection, and its inhibition can effectively restrain OSCC tumor proliferation and metastasis. Such gene may be a novel biomarker of OSCC and diagnostic tool for doctors. However, the molecular mechanism, biological behavior and its clinical application need further study and explore.

## Additional Information

**How to cite this article:** Li, G. *et al*. Prediction of biomarkers of oral squamous cell carcinoma using microarray technology. *Sci. Rep.* **7**, 42105; doi: 10.1038/srep42105 (2017).

**Publisher's note:** Springer Nature remains neutral with regard to jurisdictional claims in published maps and institutional affiliations.

## Figures and Tables

**Table 1:** A summary of OSCC microarray datasets from different GEO datasets.

	Series	Platform	Affymetrix GeneChip	References
1	GSE31056	GPL10526	[HG-U133_Plus_2] Affymetrix GeneChip Human Genome HG-U133 Plus 2 Array [Brainarray Version 12]	25
2	GSE2280	GPL96	[HG-U133A] Affymetrix Human Genome U133A Array	26
3	GSE41116	GPL5175	[HuEx-1_0-st] Affymetrix Human Exon 1.0 ST Array [transcript (gene) version]	27
4	GSE31853	GPL96	[HG-U133A] Affymetrix Human Genome U133A Array	28
5	GSE3524	GPL96	[HG-U133A] Affymetrix Human Genome U133A Array	29
6	GSE25099	GPL5175	[HuEx-1_0-st] Affymetrix Human Exon 1.0 ST Array [transcript (gene) version]	30
7	GSE30784	GPL570	[HG-U133_Plus_2] Affymetrix Human Genome U133 Plus 2.0 Array	31
